# Leveraging machine learning methods to predict post-transplant diabetes in solid organ transplant recipients

**DOI:** 10.1210/jendso/bvag236

**Published:** 2026-10-15

**Authors:** Rhea Varghese, Yingji Sun, Saba Maleki, Mamatha Bhat

**Affiliations:** Ajmera Transplant Centre, University Health Network, Toronto, ON M5G 2N2, Canada; Temerty Faculty of Medicine, University of Toronto, Toronto, ON M5S 3H2, Canada; Ajmera Transplant Centre, University Health Network, Toronto, ON M5G 2N2, Canada; Temerty Faculty of Medicine, University of Toronto, Toronto, ON M5S 3H2, Canada; Ajmera Transplant Centre, University Health Network, Toronto, ON M5G 2N2, Canada; Temerty Faculty of Medicine, University of Toronto, Toronto, ON M5S 3H2, Canada; Ajmera Transplant Centre, University Health Network, Toronto, ON M5G 2N2, Canada; Temerty Faculty of Medicine, University of Toronto, Toronto, ON M5S 3H2, Canada; Division of Gastroenterology & Hepatology, Department of Medicine, University of Toronto, Toronto, ON M5S 3H2, Canada

**Keywords:** solid organ transplant, random forest, post-transplant diabetes, logistic regression, machine learning

## Abstract

**Background:**

Post-transplant diabetes mellitus (PTDM) is a common complication following solid organ transplantation and is associated with increased morbidity and mortality. This study aimed to identify both generalizable and solid organ-specific predictors of PTDM with machine learning methods.

**Materials & Methods:**

In this study, we used 2002-2023 data from the Scientific Registry of Transplant Recipients (SRTR) to examine the risk of PTDM development across the liver (*n* = 128 016), kidney (*n* = 360 562), heart (*n* = 45 505), and lung (*n* = 36 260) transplant recipients. We examined various features at baseline using logistic regression and random forest models. Performance was evaluated using area under the receiver operating characteristic curve (AUROC), Brier scores, sensitivity, and specificity. Shapley Additive exPlanations were used for model interpretation and to identify key relevant features.

**Results:**

The incidence of PTDM ranged from 15.0% in kidney to 20.2% in lung recipients. Compared to logistic regression, random forest demonstrated modestly improved discrimination (AUROC: 0.62-0.63) and calibration (Brier: 0.124-0.156). It was found across all models that age and BMI were consistent predictors.

Prednisone and cyclosporine maintenance were often found to be associated with higher PTDM risk while mycophenolate mofetil was seen as protective. SHAP analysis demonstrated complex, nonlinear interactions and supported known risk patterns such as increased age and male sex.

**Conclusion:**

Our machine learning model identified risk factors common to post-transplant diabetes across organ transplant recipients, guiding more optimal patient survival post-transplant.

Transplantation is a critical intervention aimed at saving the lives of patients with end-stage organ disease, focusing on both short- and long-term survival (1, 2). With 1-year survival rates for the majority of solid-organ transplants surpassing 90% (kidney [97.4%], liver [92.5%], heart [91.7%], and lung [89.0%]), innovations in surgical methods, perioperative care, and immunosuppressive therapy have contributed to excellent short-term outcomes, demonstrating that transplantation still offers a significant survival benefit for all organ types (3, 4).

Post-transplant diabetes mellitus (PTDM) is a common and serious metabolic disorder that has a significant effect on long-term morbidity following solid organ transplantation (5-7). In 10-40% of transplant recipients, PTDM develops due to various diabetes risk factors, including age, obesity, and family history, along with transplant-specific factors. This condition is particularly prevalent following the administration of immunosuppressive agents, such as corticosteroids and calcineurin inhibitors (CNIs) (5, 6, 8-10). Alongside impairing quality of life, the risks of cardiovascular disease, infection, graft dysfunction, and all-cause mortality can increase following PTDM.

Although immunosuppressive agents such as calcineurin inhibitors are essential in preventing graft rejection, they have diabetogenic effects through mechanisms involving pancreatic β-cell toxicity and peripheral insulin resistance (11). In addition to CNIs, post-transplant protocols include corticosteroids, which can exacerbate glucose dysregulation by increasing hepatic gluconeogenesis and enhancing insulin resistance (12). Despite these drugs being the cause of transplant success, they strike a delicate balance between immunologic protection and metabolic disruption.

As PTDM arises from a multifactorial interplay of characteristic variables, such as age, ethnicity, genetic predisposition, pre-transplant metabolic status, and post-transplant immunosuppressive regimens, traditional statistical methods like logistic regression cannot be accurate for capturing the complex, nonlinear interactions among risk factors for PTDM (13, 14). Therefore, machine learning (ML) has emerged as a powerful tool in clinical prediction modeling, offering improved accuracy and adaptability over conventional approaches in recent years. ML algorithms, such as random forests, support vector machines, and gradient boosting techniques, have been employed to predict PTDM with promising results, leveraging large datasets to identify patterns and risk profiles that might otherwise go unnoticed (15). Baciu et al (16) and Yasodhara et al (15) have previously utilized advanced ML modalities to detect adverse outcomes across organ systems. These studies reinforce the importance of using ML tools to complement clinical judgment in transplant medicine.

In this context, machine learning offers a valuable approach to stratify PTDM risk pre- and post-transplantation, potentially guiding personalized immunosuppression regimens and closer metabolic monitoring. This study seeks to explore the use of ML-based models in predicting PTDM among solid organ transplant recipients, with the goal of enhancing early identification strategies and ultimately improving patient outcomes.

## Materials and methods

### Data source

We conducted a retrospective cohort study of solid organ recipients at all transplant centers using data from the Scientific Registry of Transplant Recipients (SRTR) between January 1, 2002, and December 31, 2023. The SRTR data system includes data on all donor, wait-listed candidates, and transplant recipients in the United States, submitted by the members of the Organ Procurement and Transplantation Network (OPTN). The Health Resources and Services Administration (HRSA), US Department of Health and Human Services provides oversight to the activities of the OPTN and SRTR contractors (17).

### Study population

Adult transplant recipients (≥18 years old) who underwent solid organ transplantation, including liver, kidney, heart, and lung transplantation from January 1, 2002 to December 2023 were included. To maintain clinical comparability and reduce confounding from age-related variations in transplant indications and outcomes, pediatric patients younger than 18 years old were not included in any analysis. Patients who underwent multi-organ transplants were also excluded from the analysis. At the time of the analysis, the dataset included 128 016 liver transplant recipients, 36 260 lung transplant recipients, 45 505 heart transplant recipients, and 360 562 kidney transplant recipients.

### Variables collected and outcomes

All variables were extracted from the SRTR database, reflecting clinical variables, such as age, gender, diabetes status pre- and post-transplant, race and ethnicity, creatinine, and BMI at the times of transplant as well as induction and maintenance of immunosuppression. For immunosuppression, variables were split into induction or maintenance therapy and further stratified by the type of immunotherapy.

In addition to individual immunosuppressive agents, we included the total number of maintenance immunosuppressive agents as a pragmatic summary measure of regimen intensity and complexity, which may independently influence metabolic risk. For maintenance 0, recipients were not on any immunosuppressive agents. Maintenance 1, 2, and 3 and above demonstrated that patients maintained on a single immunosuppressive agent, such as tacrolimus monotherapy, dual immunosuppressive therapy, commonly a calcineurin inhibitor (like tacrolimus or cyclosporine) plus another agent (like mycophenolate mofetil or steroids), and triple or more immunosuppressive agents, often tacrolimus + mycophenolate + prednisone (the classic triple regimen), or additional agents like mTOR inhibitors, respectively. Because immunosuppression data were aggregated across longitudinal patient records, recipients could contribute to multiple indicators as their regimens changed over time. These indicators were therefore analyzed separately rather than as mutually exclusive categories. Post-transplant diabetes mellitus (PTDM) was defined using SRTR follow-up data indicating diabetes diagnosed after transplantation among recipients without pre-transplant diabetes. The outcome was assessed within 10 years following transplantation. Individuals with documented pre-transplant diabetes were excluded from the PTDM outcome definition.

### Machine learning models

#### Model training, evaluation, and interpretability

In order to evaluate prediction performance, the dataset was randomly divided into 70% for model training and 30% for testing. We put a number of classification methods into practice, such as random forest and logistic regression (with and without SMOTE and threshold adjustment), to assess how well they predicted post-transplant diabetes mellitus across a range of clinical and immunological factors. We optimized important parameters like the number of trees, maximum depth, and minimum samples per split for random forests, and regularization strength for logistic regression using 5-fold cross-validation within the training set to ensure model robustness and reproducibility.

#### Machine learning classification model

By leveraging the demographic data from the SRTR database, we developed ML techniques to predict the PTDM in all solid organ transplant types. Key demographic and clinical variables were age, gender, race, body mass index (BMI), international normalized ratio (INR), bilirubin, creatinine, model for end-stage liver disease (MELD), and comprehensive information on induction and maintenance of immunosuppressive regimens. The type and intensity of immunosuppressive regimens were studied individually for induction therapy and in 4 groups for maintenance therapy.

By utilizing logistic regression models (LR) (with and without SMOTE and threshold adjustment) and random forest (RF) models, linear and non-linear associations between these predictors and the risk of PTDM could be captured. Ensemble methods such as RF improve predictive robustness by aggregating multiple trees, diminishing overfitting, and detecting the complex interactions among demographic, biochemical, and immunosuppressive variables.

To further clarify the relative contribution of each feature, we subsequently applied SHAP (SHapley Additive exPlanations) analyses. This approach provided interpretable insights into which clinical factors most strongly influenced PTDM risk.

This ML framework enables precise risk stratification and prediction of PTDM by providing comprehensive insights for tailoring immunosuppressive therapy and improving post-transplant management to optimize patient outcomes.

### Statistical analysis

Continuous variables are presented as mean ± SD, and categorical variables as number (percentage). *P*-values reflect comparisons across groups using ANOVA for continuous variables and chi-square tests for categorical variables. Any variables with missingness over 20% were dropped. To further address missing data, we applied different imputation strategies based on variable types. Variables with >20% missing data were excluded from the analysis. For the remaining variables, missing values were handled according to variable type. Continuous variables were imputed using the mean of the observed values, whereas categorical variables were imputed using the mode. Where a clinically meaningful “missing” or least informative category was available, missing categorical values were instead assigned to this category to avoid introducing assumptions about the underlying value. The imputation approach was selected based on the variable's measurement scale, distribution, and clinical context, with the goal of maintaining sample size while using simple and interpretable methods appropriate for a large-scale clinical dataset. The proportion of missing data for individual variables and the number of observations affected by each imputation strategy were not reported, which limits assessment of the potential impact of missing-data handling on the results and is acknowledged as a limitation.

Performance was assessed based on key metrics, including area under the receiver operating characteristic curve (AUROC), sensitivity, and specificity with corresponding 95% CI. To enhance model interpretability and support clinical translation, we conducted SHAP (SHapley Additive exPlanations) analysis (18). At the population level, Shapley values enabled us to identify the most influential predictors driving overall model performance. This helped generate clinically meaningful insights into risk stratification and potential intervention targets across the cohort.

### Code availability

All analyses were performed using Python 3.10.14. We used Pandas (v2.2.2) and NumPy (v2.0.1) for data preprocessing, cleaning, and numerical operations. ML model development, training, and evaluation, such as logistic regression (with and without SMOTE and threshold adjustment) and random forest, were done by using scikit-learn (v1.5.1). The SHAP library (v0.42.0) was used for conducting model interpretability.

## Results

This study included 570 343 solid organ recipients, including 63.22% kidney transplant, 22.45% liver transplant, 6.36% lung transplant, and 7.98% heart transplant. The mean (SD) age for each group was 51.3 (13.7), 54.3 (10.9), 55.9 (13.0), and 52.8 (12.8), respectively. 60.8%, 34.2%, 59.1%, and 25.7% were female in each group, respectively. Other demographic features of this study were demonstrated in Table 1.

**Table 1:** Baseline characteristics of transplant recipients by organ type

Variable	Kidney (*n* = 360 562)	Liver (*n* = 128 016)	Lung (*n* = 36 260)	Heart (*n* = 45 505)	*P*-value
Age, mean (SD)	51.3 (13.7)	54.3 (10.9)	55.9 (13.0)	52.8 (12.8)	<.001
Female sex, *n* (%)	219 315 (60.8)	43 784 (34.2)	21 433 (59.1)	11 698 (25.7)	<.001
BMI (kg/m^2^), mean (SD)	27.9 (5.5)	28.4 (5.9)	25.3 (4.7)	27.2 (5.0)	<.001
White race, *n* (%)^a^	122 982 (34.1)	18 383 (14.4)	4141 (11.4)	11 751 (25.8)	<.001
Black race, *n* (%)^a^	266 166 (73.8)	116 875 (91.3)	33 108 (91.3)	35 757 (78.6)	<.001
Asian race, *n* (%)^a^	338 584 (93.9)	122 557 (95.7)	35 540 (98.0)	44 019 (96.7)	<.001
Creatinine, mean (SD)	3.2 (2.8)	1.6 (1.4)	—	—	—
PTDM, *n* (%)	54 249 (15.0)	24 451(19.1)	7314 (20.2)	8391 (18.4)	
0 Maintenance drugs, *n* (%)^a^	4304 (1.2)	569 (0.4)	288 (0.8)	242 (0.5)	.002
1 Maintenance drug, *n* (%)^a^	354 782 (98.4)	122 148 (95.4)	35 979 (99.2)	45 117 (99.1)	.679
2 Maintenance drugs, *n* (%)^a^	256 084 (71.0)	91 232 (71.3)	33 588 (92.6)	41 365 (90.9)	<.001
≥3 Maintenance drugs, *n* (%)^a^	114 562 (31.8)	43 221 (33.8)	3241 (8.9)	4770 (10.5)	<.001

Data presented as mean ± SD or *n* (%). *P*-values reflect comparisons across organ types using ANOVA or chi-square tests as appropriate.

^*a*^Race categories were not mutually exclusive. Immunosuppressive medication data were aggregated across longitudinal patient records, with individuals potentially contributing to multiple categories as their treatment regimens changed over time. Consequently, percentages may sum to greater than 100%.

## Model performance and interpretability

Table 2 demonstrates the performance of LR, logistic regression with SMOTE, threshold-adjusted logistic regression, and random forest classifiers across liver, kidney, lung, and heart transplant cohorts. Model discrimination, which is assessed by AUROC, was modest across all methods and organs, reflecting the complexity of predicting PTDM from clinical and immunologic data alone. RF models indicated AUROC scores that ranged from 0.62 for liver and lung to 0.63 for heart and kidney and moderate discriminative performance.

**Table 2:** Comparative performance of machine learning and logistic regression models for predicting post-transplant diabetes mellitus across solid organ transplant cohorts

	Measure	Liver	Kidney	Lung	Heart
LR (best param)	AUROC	0.579	0.615	0.608	0.615
	Sensitivity	53.3%	57.5%	50.7%	45.2%
	Specificity	57.8%	57.7%	65.1%	70.8%
LR (SMOTE)	AUROC	0.577	0.615	0.539	0.615
	Sensitivity	53.8%	57.7%	50.6%	46.1%
	Specificity	56.8%	56.4%	64.6%	69.5%
LR (adjusted threshold)	AUROC	0.553	0.561	0.539	0.546
	Sensitivity	53.8%	89.7%	87.5%	87.7%
	Specificity	56.8%	22.6%	20.3%	21.5%
Random forest	AUROC	0.62	0.63	0.62	0.63
	Sensitivity	57.6%	57.2%	44.2%	43%
	Specificity	59.8%	60.7%	75.3%	76.6%

### Logistic regression (best parameters & SMOTE)

For logistic regression (best parameters), AUROC values indicated 0.579 and 0.608 in liver and lung recipients, while kidney and heart recipients had 0.615, which is slightly better; however, they still show modest performance. While specificity ranged from 57.7% in kidney recipients to 70.8% in heart recipients, sensitivity was highest in kidney recipients (57.5%) and lowest for heart recipients (45.2%).

Performance was similar to the baseline logistic regression when class imbalance was addressed using SMOTE. There was minimal variation in sensitivity and specificity amongst organ types, and AUROC values were almost unchanged (0.577-0.615).

### Threshold adjustment

Threshold adjustment significantly improved sensitivity in all organs, particularly in recipients of the kidney (89.7%), lung (87.5%), and heart (87.7%). Despite this improvement, the specificity was significantly reduced (22.6-21.5%). The corresponding AUORC values for the liver, kidney, lung, and heart are 0.520, 0.561, 0.539, and 0.546.

### Random forest model

With AUROC values ranging from 0.62 to 0.63 for all organ types, the random forest model performed slightly better overall and maintained a better balance between sensitivity and specificity. For the kidney, sensitivity was 57.2% and specificity was 60.7%. Liver, sensitivity 57.6%, specificity 59.8%. Lung, sensitivity 44.2%, specificity 75.3% and heart, sensitivity 43%, specificity 76.6%.

## Solid organ recipients characteristics with PTDM

### Clinical characteristics and feature importance based on SHAP analysis in kidney transplant recipients

A total 54 249 of 360 562 (15.0%) of kidney recipients had PTDM (Tables 1 and 3), Age, BMI, race (particularly greater among the black patient group), and the usage of tacrolimus and MMF all showed statistically significant differences (19).

**Table 3:** Baseline characteristics of PTDM vs non-PTDM patients by transplant type

Variable	Kidney PTDM−	Kidney PTDM+	Liver PTDM−	Liver PTDM+	Lung PTDM−	Lung PTDM+	Heart PTDM−	Heart PTDM+
*n*	306,313	54,249	103,565	24,451	28,946	7,314	37,114	8,391
Creatinine, mean (SD)	3.2 (2.8)	3.0 (2.6)	1.6 (1.4)	1.6 (1.4)	—	—	—	—
Sodium, mean (SD)	—	—	135.9 (5.0)	135.9 (4.6)	—	—	—	—
MELD, mean (SD)	—	—	19.2 (9.6)	18.2 (8.9)	—	—	—	—
Bilirubin, mean (SD)	—	—	8.6 (10.8)	8.0 (10.5)	—	—	—	—
INR, mean (SD)	—	—	1.9 (1.0)	1.8 (0.9)	—	—	—	—
BMI (kg/m²), mean (SD)	27.7 (5.5)	28.9 (5.4)	28.3 (5.9)	28.8 (5.7)	25.3 (4.6)	25.4 (4.8)	27.1 (5.0)	27.9 (5.0)
Age, mean (SD)	50.8 (13.9)	53.8 (11.7)	54.1 (11.2)	55.0 (9.6)	56.4 (12.8)	54.2 (13.5)	52.6 (13.1)	53.3 (11.5)
Female sex, *n* (%)	185,316 (60.5%)	33,999 (62.7%)	36,185 (34.9%)	7,599 (31.1%)	17,107 (59.1%)	4,326 (59.1%)	9,766 (26.3%)	1,932 (23.0%)
Asian race, *n* (%)	18,899 (6.2%)	3,079 (5.7%)	4,356 (4.2%)	1,103 (4.5%)	590 (2.0%)	130 (1.8%)	1,237 (3.3%)	249 (3.0%)
Black race, *n* (%)	78,237 (25.5%)	16,159 (29.8%)	8,660 (8.4%)	2,481 (10.1%)	2,467 (8.5%)	685 (9.4%)	7,880 (21.2%)	1,868 (22.3%)
White race, *n* (%)	203,560 (66.5%)	34,020 (62.7%)	89,055 (86.0%)	20,578 (84.2%)	25,677 (88.7%)	6,442 (88.1%)	27,576 (74.3%)	6,178 (73.6%)
Multiracial, *n* (%)	1,648 (0.5%)	211 (0.4%)	510 (0.5%)	98 (0.4%)	80 (0.3%)	22 (0.3%)	174 (0.5%)	34 (0.4%)
Indigenous, *n* (%)	2,665 (0.9%)	561 (1.0%)	821 (0.8%)	143 (0.6%)	110 (0.4%)	28 (0.4%)	110 (0.3%)	31 (0.4%)
Pacific Islander, *n* (%)	1,301 (0.4%)	219 (0.4%)	163 (0.2%)	48 (0.2%)	21 (0.1%)	7 (0.1%)	137 (0.4%)	31 (0.4%)
Non-White, *n* (%)	306,313 (100.0%)	54,249 (100.0%)	103,565 (100.0%)	24,451 (100.0%)	28,946 (100.0%)	7,314 (100.0%)	37,114 (100.0%)	8,391 (100.0%)
0 Maintenance drugs, *n* (%)	3,585 (1.2%)	719 (1.3%)	451 (0.4%)	118 (0.5%)	222 (0.8%)	66 (0.9%)	181 (0.5%)	61 (0.7%)
1 Maintenance drug, *n* (%)	301,391 (98.4%)	53,391 (98.4%)	98,873 (95.5%)	23,275 (95.2%)	28,727 (99.2%)	7,252 (99.2%)	36,807 (99.2%)	8,310 (99.0%)
2 Maintenance drugs, *n* (%)	216,858 (70.8%)	39,226 (72.3%)	74,137 (71.6%)	17,095 (69.9%)	26,809 (92.6%)	6,779 (92.7%)	33,653 (90.7%)	7,712 (91.9%)
≥3 Maintenance drugs, *n* (%)	97,962 (32.0%)	16,600 (30.6%)	34,571 (33.4%)	8,650 (35.4%)	2,578 (8.9%)	663 (9.1%)	3,949 (10.6%)	821 (9.8%)

Data are presented as mean (SD) or n (%). Race categories were represented using separate binary indicators and were not necessarily mutually exclusive. Immunosuppressive medication data were aggregated across longitudinal patient records, with individuals potentially contributing to multiple maintenance-drug categories as their treatment regimens changed over time. Consequently, percentages may sum to greater than 100%.

*Note to editor: The original descriptive tables inadvertently reported the frequency of non-membership (coded as 1) rather than membership (coded as 0) for each racial category. This has been corrected in Tables 1 and 3 by reversing the binary indicators and recalculating both the absolute counts and corresponding percentages. All corrected percentages were checked against their respective cohort denominators. This was a descriptive reporting correction and did not alter the underlying patient counts or cohort sizes.

The kidney logistic regression was shown in Fig. 1A (Brier = 0.238). While age, BMI, and creatinine were associated with PTDM, race and sex variables had minimal impact on the plot; nevertheless, being Black caused a slight positive shift in SHAP values. Prednisone or cyclosporine maintenance was linked to an increased risk of PTDM, but induction was generally protective.

**Figure 1:**
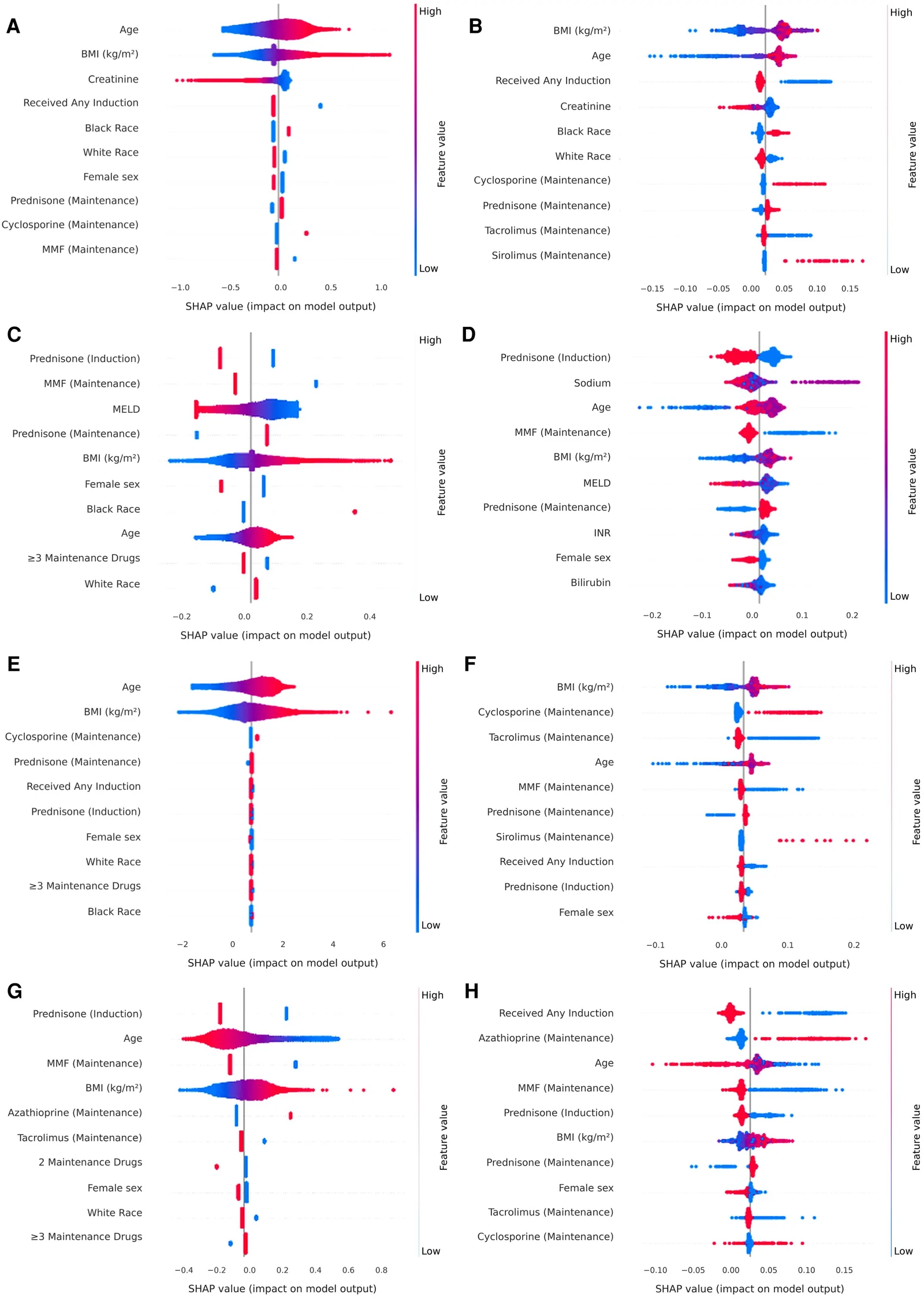
SHAP summary plots of feature contributions to post-transplant diabetes mellitus (PTDM) risk across organs and models. Feature importance values (SHAP) indicate the direction and magnitude of each feature's contribution to model predictions for PTDM. (A) Kidney logistic regression. (B) Kidney random forest. (C) Liver logistic regression. (D) Liver random forest. (E) Heart logistic regression. (F) Heart random forest. (G) Lung logistic regression. (H) Lung random forest.

Within the kidney demographic, the random forest model (brier score = 0.124) found BMI and age to be the strongest predictors, with them being correlated with increased PTDM risk (Fig. 1B). Receiving any induction therapy was protective as were higher creatinine levels. Being Black had a modest positive relationship with PTDM while being of White race was more protective. The maintenance usage of cyclosporine and prednisone was positively correlated with PTDM in terms of immunosuppressants.

### Clinical characteristics and feature importance based on SHAP analysis in liver transplant recipients

In the liver transplant group (128, 016), PTDM developed in 19.1% of recipients (Table 1). The mean age for the PTDM group was 54.1 (11.2). Moreover, these recipients had higher BMI and lower MELD scores at the time of transplant, potentially reflecting more severe liver dysfunction. Several immunosuppressive agents showed statistically significant differences in usage between PTDM and non-PTDM groups (19).

For the LT recipients, both the RF model and LR identified using prednisone as an inductive agent was the most influential PTDM predictor with it being protective of PTDM (Fig. 1C and 1D). The RF demonstrated that higher BMI and older age had a modest positive contribution to PTDM development. While using prednisone as maintenance increased the risk, administering MMF as maintenance was protective.

In contrast to the RF, the logistic regression SHAP values emphasized the demographic and categorical variables. Black race and being of male sex were positively correlated with higher PTDM risk. However, the white race was less positively correlated with a higher risk of PTDM. Both brier scores (RF of 0.153 and LR of 0.245) reinforce good probabilistic calibration. Overall, both models reinforce the importance of maintenance of immunosuppression, corticosteroid exposure, and metabolic risk factors such as BMI in contributing to PTDM risk.

### Clinical characteristics and feature importance based on SHAP analysis in heart transplant recipients

In the heart transplant group (45, 505), PTDM incidence was 18.4% and skewed toward older age, higher BMI and being of female sex (*P* = <.05, Tables 1 and 3). In the heart logistic regression model (Brier score = 0.235), it was found that age was the most predictive feature for PTDM (Fig. 1E). An elevated BMI was also a positive contributor to PTDM, suggesting metabolic risk factors were predictive. Cyclosporine and prednisone (maintenance therapies) were associated with higher SHAP values (19). Contrastingly, the RF model (Brier score = 0.145) had a more distributed feature influence. BMI once again played a dominant role, and cyclosporine and sirolimus (maintenance) were also highly associated with PTDM. Interestingly, Tacrolimus and MMF as a maintenance drug had a weaker and more neutral SHAP affect veering on protective (Fig. 1F). Calibration of both models demonstrated a reasonable level of concordance between predicted and observed probabilities.

### Clinical characteristics and feature importance based on SHAP analysis in lung transplant recipients

Among lung transplant recipients (*n* = 36, 260), 20.2% developed PTDM (Table 1). Age was a strong differentiator (*P* < .001), whereas BMI did not have significantly difference.

In lung transplant recipients, LR demonstrated that prednisone induction therapy was protective against PTDM. Being on Azathioprine increased the risk of PTDM while being on mycophenolate mofetil was protective (Fig. 1G). Moreover, younger age was correlated with a higher risk of PTDM. MMF maintenance was protective against PTDM, while azathioprine was positively associated with PTDM (19). BMI (kg/m^2^) was also positively associated with PTDM development, reinforcing obesity as a known modifiable risk factor. In the same population, RF indicated the additional contributors included prednisone (induction as protective, maintenance as risk) (Fig. 1H). Notably, female sex was associated with being protective. The Brier score for the calibrated model was 0.156 and 0.238 indicating good probabilistic calibration.

## Discussion

In this large multi-organ analysis using national transplant registry data, we both developed and evaluated ML models to predict PTDM across kidney, liver, heart, and lung transplant recipients. Traditional statistical comparisons such as *P* values were complemented with SHAP analysis to better elucidate directionality and relative importance. While the overall model performance was modest, with AUROCs ranging from 0.58 to 0.63, several meaningful patterns emerged that explore insight into both shared and organ-specific PTDM risk factors. Across organ cohorts, random forest models showed somewhat better discrimination and lower Brier scores than logistic regression, although these changes were not very noticeable. This demonstrates that using only baseline clinical characteristics results in low prediction performance. Though the Brier score was applied as a global indicator of predictive performance, it would reflect both calibration and discrimination. These results indicate that complex and dynamic factors that were not fully captured at baseline affect PTDM and that adding longitudinal and time-dependent variables may be recommended to enhance model performance.

This study enforces that ML can improve interpretability but can remain limited in discrimination. RF classifiers outperformed LR in both discriminations, as reflected in AUROC scores, and calibration as indicated by lower Brier scores, especially in the kidney and heart cohorts. However, this improvement was modest, and even the best performing models only demonstrated moderate predictive capacity. This underscores the inherent complexity of transplant medicine and PTDM development, where intricate interactions extend beyond readily available clinical and immunologic variables captured at the baseline. Nevertheless, calibration metrics were acceptable, and the SHAPley analyses yielded clinically plausible relationships, further supporting machine learnings’ ability to elucidate clinicians in risk stratification. The moderate performance highlights an important reality that PTDM is a multifactorial outcome affected by dynamic, post-transplant factors not captured in the baseline data alone.

Across all organ types examined, BMI consistently emerged as a strong positive predictor of PTDM development, reinforcing metabolic risk factors as a key PTDM contributor. Age was generally positively linked with PTDM risk across multiple organ transplants, consistent with established mechanisms including beta-cell senescence and age-related insulin resistance (20-23). However, in lung transplant recipients, younger age was associated with higher PTDM risk. Since younger recipients may undergo more aggressive or prolonged immunosuppression, especially corticosteroids or calcineurin inhibitors due to a presumed greater physiologic reserve and tolerance for intensive regimens (24-27). Additionally, high early tacrolimus levels have been significantly associated with PTDM in lung cohorts (12, 28). Younger lung transplant recipients are at higher risk of abnormal glucose metabolism and pancreatic insufficiency primarily due to the high prevalence of cystic fibrosis and interstitial lung (29-31).

Prednisone was repeatedly a feature of importance across all organs, both as maintenance and induction therapy. Interestingly, we observed a consistent pattern whereby prednisone induction was associated with protection against PTDM, while prednisone maintenance therapy was associated with increased PTDM risk. When used during the initial post-transplant phase, prednisone induction therapy can help control the initial inflammatory response and reduce early rejection, which can trigger metabolic derangements (32). Meanwhile, long-term maintenance corticosteroids therapy can have metabolic side effects due to its interference with insulin sensitivity and glucose metabolism, leading to hyperglycemia and ultimately PTDM (12). This discrepancy aligned with prior studies suggesting that acute high dose corticosteroid pulses may induce stress-related hyper-glycemia that resolves, while chronic exposure promotes adiposity, insulin resistance, and PTDM development. This, however, does exist as a limitation as well as the leading evidence indicates that steroids generally increase the risk of PTDM (33, 34).

### Kidney transplant recipients

Within the kidney population, the use of cyclosporine and sirolimus as maintenance therapy was both correlated with an increased PTDM risk, while induction therapy was generally protective. Diabetogenic profiles of calcineurin inhibitors and mTOR inhibitors supported these findings.

Interestingly, higher creatinine levels appeared inversely associated with PTDM risk, contrary to clinical expectations. Impaired renal function is a known contributor to insulin resistance (35). Since all variables in this study, including creatinine, reflect baseline values at the time of transplant, this may not represent a true causal relationship but rather a surrogate or other perioperative factors. Recipients with lower creatinine at baseline may represent a population with better overall metabolic reserve or younger age. This may lead to more aggressive or prolonged immunosuppression (36). Conversely, patients with elevated baseline creatinine may have received more conservative immunosuppression protocols. Our findings indicate the importance of temporal context when attributing feature attribution and indicate future models can benefit from incorporating time updated laboratory values.

### Liver transplant recipients

Within the liver cohort, the MELD score was negatively correlated with PTDM, a seemingly paradoxical finding given that higher MELD scores typically indicate more severe liver dysfunction. However, this may reflect important selection and treatment biases within liver transplantation. Patients with lower MELD scores are often transplanted for hepatocellular carcinoma under exception criteria rather than for decompensated liver disease. These patients often have preserved liver function but tend to be older and have higher baseline metabolic risk factors such as obesity and prediabetes, all of which contribute to PTDM development (37, 38). MMF maintenance was seen to be mildly protective, which aligns with prior knowledge that MMF and azathioprine lack a significant metabolic adverse event compared to CNIs (39).

### Heart transplant recipients

Within the heart transplant cohort, cyclosporine maintenance was mildly associated with higher PTDM risk, while tacrolimus demonstrated a protective effect. These findings align with prior studies showing that among heart transplant recipients, tacrolimus-based regimens resulted in less hyperglycemia compared to cyclosporine-based protocols (40). This organ-specific trend could be the result of variations in drug monitoring procedures, immunosuppressive regimens, or pharmacokinetic profiles unique to the heart transplant cohort.

### Lung transplant recipients

Among lung recipients’ maintenance therapy with MMF and tacrolimus was associated with a lower PTDM risk while azathioprine was linked to increased risk. The protective association with MMF is consistent with renal transplant data demonstrating a 16-22% reduction in PTDM risk with MMF compared to azathioprine (41). While tacrolimus is recognized as a diabetogenic immunosuppressant, lung cohorts have indicated variability in this association, with potential protective effects when used in conjunction with steroid-sparing strategies. Induction therapy in general was considered protective, a finding consistent with prior research that found lower early PTDM rates followed induction-based immunotherapy (40).

### Clinical implications

Despite modest predictive performance, these findings may have clinical relevance as risk stratification tools rather than standalone decision-making instruments. The models may help identify transplant recipients at relatively higher risk of PTDM who could benefit from closer metabolic monitoring, earlier lifestyle interventions, and more frequent glycemic screening, particularly in the early post-transplant period.

These models should not be interpreted as ready for direct clinical decision-making. Their utility lies primarily in risk stratification and hypothesis generation, rather than guiding individualized treatment. The absence of time-dependent variables, such as longitudinal glucose measurements, immunosuppressive dose adjustments, and dynamic clinical changes, likely limits predictive performance and clinical applicability.

Importantly, these findings reinforce the role of baseline metabolic risk factors and immunosuppressive exposures in shaping PTDM risk across organ types. At a population level, such models may help identify higher-risk subgroups and inform targeted surveillance strategies. However, given the limited sensitivity and specificity, these models are not currently suitable for guiding individualized treatment decisions or modifying immunosuppressive regimens in isolation.

## Limitations

This study has several limitations. While the SRTR dataset is extensive and nationally representative, it lacks key features relevant to PTDM prediction, including family history of diabetes, HbA1c values, fasting glucose levels, detailed pre-transplant metabolic assessment, and information on post-transplant diabetes management (eg, insulin vs oral antihyperglycemic agents). The modest differentiation observed may be partially explained by the absence of these established diabetes risk factors, which likely limited model performance.

The data used for model development was limited to baseline features at transplantation without any longitudinal data, such as changes in lab values, immunosuppressive regimens, dose adjustments, rejection episodes, or infections, and so on, all which can modify PTDM risk. This static analysis may obscure more dynamic processes underlying PTDM. We also observed substantial missingness in some features, which also led to the inclusion and exclusion of certain features. Missing data may have introduced bias and reduced the robustness of our findings. Variables with >20% missingness were excluded, while missing values in retained variables were addressed using predefined imputation strategies based on variable type. However, because the extent and patterns of missingness across individual variables were not systematically quantified or reported, we cannot exclude the possibility that missingness was non-random and may have influenced the observed associations. Additionally, mean and mode imputation may underestimate variability and introduce bias when the proportion or pattern of missing data is substantial. These limitations should be considered when interpreting the results. The SRTR data are also US specific, so the findings may not generalize to other healthcare systems, or the international population where transplant protocols, immunosuppression practices, screening approaches, and PTDM management strategies may differ substantially.

While there were attempts made to address the class's imbalance via SMOTE and adjusting the threshold, the overall model of discrimination remains modest. This suggests that baseline data alone may be insufficient to accurately predict PTDM and underscore the multifactorial complexity inherent in transplant medicine. Given the modest nature of this tool, it requires further testing and validation before being deployed for direct clinical decision-making.

In terms of feature selection, our finding of inductive prednisone is viewed as a limitation. As corticosteroids are a recognized dose- and duration-dependent cause of diabetes, our findings regarding induction dosing should be viewed as hypothesis-generating and interpreted within the context of concurrent maintenance immunosuppression (42, 43). Furthermore, SHAP values reflect a feature's marginal contribution to the model's predictions rather than an isolated causal effect; because PTDM is multifactorial, driven by interacting contributions from recipient age, calcineurin inhibitor choice and exposure, BMI, and cumulative steroid burden, the importance attributed to induction dosing may partly capture correlated maintenance-era exposures rather than an independent protective effect (44, 45).

Finally, this study was of a retrospective design and cannot establish causal relationships. The models would require validation prospectively in external datasets and would require the inclusion of post-transplant time-varying features to enhance generalizability and clinical utility.

## Conclusions

In this multicenter analysis of US transplant recipients with the SRTR dataset, we evaluated the utility of various machine learning approaches including random forests and logistic regressions with SHAPley analyses to predict post-transplant diabetes mellitus across kidney, liver, heart, and lung cohorts.

Our findings demonstrated both shared as well as organ specific PTDM risk features. BMI, age, and prednisone were found to be highly linked to PTDM across all organs. Other immunosuppressants were found to have various associations with PTDM depending on the organ and model used, which highlights the complex interactions at play. Random forests generally demonstrated modest discrimination and better calibration than logistic regressions (AUROC 0.61-0.63), but overall predictive performance was limited due to a lack of longitudinal data and missingness in the data. SHAPley analyses bolstered model transparency and elucidated clinically coherent associations that can help inform risk stratification. For future directions, efforts should further involve dynamic and time varying features such as glucose trends and cumulative immunosuppression as well as more complex machine learning models.

## Data Availability

The dataset with the data collected for this study is available from the authors upon reasonable request. The data reported here have been supplied by the Hennepin Healthcare Research Institute as the contractor for the Scientific Registry of Transplant Recipients (SRTR). The interpretation and reporting of these data are the responsibility of the author(s) and in no way should be seen as an official policy of or interpretation by the SRTR or the US Government.

## Abbreviations

AUROC: area under the receiver operating characteristic curve
BMI: body mass index
CNIs: calcineurin inhibitors
INR: international normalized ratio
LR: logistic regression
MELD: model for end-stage liver disease
MMF: mycophenolate mofetil
ML: machine learning
OPTN: organ procurement and transplantation network
PTDM: post-transplant diabetes mellitus
RF: random forests
SRTR: scientific registry of transplant recipients
